# The PHA Test as an Indicator of Phagocytic Activity in a Passerine Bird

**DOI:** 10.1371/journal.pone.0084108

**Published:** 2013-12-31

**Authors:** Concepción Salaberria, Jaime Muriel, María de Luna, Diego Gil, Marisa Puerta

**Affiliations:** 1 Department of Evolutive Ecology, Museo Nacional de Ciencias Naturales (Centro Superior de Investigaciones Científicas), Madrid, Spain; 2 Department of Animal Physiology, Facultad de Ciencias Biológicas (Universidad Complutense de Madrid), Madrid, Spain; CNRS, Université de Bourgogne, France

## Abstract

Several techniques in ecological immunology have been used to assess bird immunocompetence thus providing useful information to understand the contribution of the immunological system in life-history decisions. The phytohaemagglutinin (PHA)-skin test has been the most widely employed technique being interpreted as the sole result of T lymphocytes proliferation and hence used to evaluate acquired immunological capacity. However, the presence of high numbers of phagocytic cells in the swelling point has cast some doubt about such an assumption. To address this issue, we collected blood from 14 days-old nestlings of spotless starling (*Sturnus unicolor*), administered subcutaneous PHA immediately after and then measured the swelling response 24 hours later. Differential counts of white blood cells suggested that an intense development of acquired immunological defences was taking place. The phagocytic activity of both heterophiles and monocytes was also very intense as it was the swelling response. Moreover, our results show, for the first time in birds, a positive relationship between the phagocytic activity of both kinds of cells and the swelling response. This broadens the significance of the PHA test from reflecting T lymphocytes proliferation -as previously proposed but still undetermined *in vivo*- to evaluate phagocytosis as well. In other words, our data suggest that the PHA swelling response may not be considered as the only consequence of processes of specific and induced immunity –T lymphocytes proliferation- but also of constitutive and nonspecific immunity –heterophiles and monocytes phagocytosis. We propose the extensive use of PHA-skin test as an optimal technique to assess immunocompetence.

## Introduction

Life history theory explains the decisions taken by organisms to optimize their survival and reproduction due to ecological challenges imposed by natural selection and other evolutionary forces [1]–[3]. Evolutionary ecologists have long been interested in the effects of life-history decisions on organisms' immunocompetence, resulting in a new field denominated immunoecology, where birds have been the main model. Immunocompetence, defined as the capacity of an individual to mount an appropriate immune response following the exposure to a pathogen, is a critical aspect of disease resistance and survival [4]. Since immunological responses are energy consuming [5], [6], the organism dealing with an immunological challenge also faces the need of distributing its energy budget among the different systems and functions that allow survival. Accordingly, by evaluating the functional potential of its immunological system, the quality of an individual can also be inferred. Hence, there is increasing evidence that immune function may constitute an important determinant of fitness [7], [8]. Therefore, assessment of immunocompetence in free-living individuals is emerging as an important tool in evolutionary and ecological research. However, the types of assays that can be employed with wild animals are often constrained by the stress that results from capture and handling, the unreliability of recapturing animals, the prohibition of terminal studies, the lack of specialized reagents, and the small size of many study species [9].

Given both the presence of multiple components of the immunological system in blood and the easiness of getting a blood sample, field researchers commonly evaluate immune components in blood as a tool to assess the quality of an individual or a population in both environmental physiology and ecology studies. Several parameters reflecting different components of the whole system have been used to draw plausible conclusions (reviewed by Demas et al. [4]). The vertebrate immunological system gives both innate and acquired responses, the former considered constitutive and unspecific in contrast with the latter, considered specific and induced- in the defence component model proposed by Schmid-Hempel and Ebert [10]. Since any immunological challenge activates the innate component of the immunological system as a first response, functional capacities of phagocytic cells have been assayed in either whole blood samples or in preparations of isolated cells to assess the state of the innate response [11]. On the contrary, acquired immunity develops with lymphocytes proliferation. A classical tool to assess acquired immunological capacity *in vivo* has been the delayed-type hypersensitivity [12], [13]. It is based on the fact that the first exposure to an antigen recruits cell components of the innate system that initiate the acquired response, with a proliferation of lymphocytes. A second exposure several days later results in an intense swelling in the injection point [14] that is used as an index of acquired immune response. However, this has the drawback than animals need to be captured at least three times. The most widely used alternative for evaluating cell-mediated acquired immunity in immunoecology is the phytohaemagglutinin (PHA) swelling test that only involves two captures separated by 24 hours [15]. Several studies have shown a positive relationship between PHA response and several aspects of individualś fitness such as condition [16], [17] or adult survival probability [18], [19]. For these reasons PHA response has been regarded as a reliable generic surrogate for disease resistance [20]. PHA test rests on the fact that many T-cells are responsive to PHA, so that PHA produces T lymphocyte proliferation directly without the need of antigen presenting cells [14]. This, together with the fact that thymectomy reduces not only lymphocytes number but also the *in vivo* response to PHA [21] have led field biologists to interpret the PHA swelling response as representative of the acquired immune capacity of an animal [8], [22]–[25]. Nonetheless, PHA not only produces T-lymphocytes proliferation but also agglutinate blood cells by binding membrane components which attract innate components of the immunological system so that there are discrepancies about recognizing the PHA-swelling response as solely a T-cell mediated response [26], [27]. Recently, new evidences have shown the presence of cell components of the innate immunological system in the point of PHA injection. In fact, during the 24 hours post injection, the number of macrophages, heterophiles and basophiles increase 2–5 times whereas that of lymphocytes does not increase significantly [28]. Moreover, the molecular form of PHA that does not produce lymphocytes proliferation but only blood cells agglutination produces a larger swelling reaction than that achieved with the molecular form producing proliferation [29]. Accordingly, several authors underlines that the 24 hours PHA swelling reaction is dynamic and involves both innate and acquired components so that it cannot be considered as representative of T-cell proliferation [20], [28], [29].

In this study we looked for additional functional evidence showing the contribution of the innate immunological system to the PHA swelling response [20], [29]. Accordingly, we related the PHA swelling response to the functional activity of the phagocytic cells in blood, namely, heterophiles and monocytes. In the spring of 2009, we collected blood from nestlings of spotless starling and, after blood collection, we injected PHA to assess swelling 24 hours later. Fresh blood samples allowed us to measure separately the phagocytic activity of heterophiles and monocytes and relate it to the swelling response obtained 24 hours later. We also studied the cell composition of those blood samples since haematological studies have shown that blood cells experience a process of perinatal maturation so that the presence of red blood cells (RBC) and white blood cells (WBC) changes in number and in capabilities along this period [30], [31].

## Materials and Methods

### Animals, study area and blood collection

This study was conducted during the 2009 reproductive season in Soto del Real, Madrid (central Spain), where a colony of spotless starlings (*Sturnus unicolor*) have been breeding in nest boxes since 2002. The facultative polygynous spotless starling is a dimorphic species in plumage and size (with males being the larger sex). Females lay four or five eggs per clutch and can breed once or twice in a season (first and second clutches), sometimes laying a replacement clutch when another is lost. Incubation is usually performed by females and males vary considerably in the amount of parental care they provide [32], [33]. The nestlings are altricial and stay in the nests for 25 days. Nests were visited every day to determine laying and hatching date. After measuring body weight, a blood sample was collected from the jugular vein of 14 days- old- chicks in heparinised syringes. Immediately after blood collection, PHA was injected and swelling response was measured 24 h later (see below). Blood was transported to the lab in cool containers when fresh counting took place no more than 6 hours after collection. 31 nestlings (16 males, 12 females and 3 undetermined sex) were used for cell counting, phagocytosis test and swelling response (some samples were lost on processing). No more than one chick per nest was considered, so that no brothers were analysed. The protocol of work used in this research was approved by the Environment Department of the Autonomous Community of Madrid. Permission to work in the study area was granted by the Ayuntamiento de Soto del Real and the Consejería de Medio Ambiente of the Comunidad Autónoma de Madrid.

### Cell counting

On arrival to the lab, blood samples were gently but intensively agitated to obtain a uniform distribution of blood cells. Aliquots of blood were diluted in haematological pipettes with Natt and Herrick's solution (200 and 50 times for RBC and WBC, respectively). A Thoma chamber was used for cell counting. All the large squares and at least 48 small ones were counted for RBC and WBC, respectively.

Blood smears were fixed by a 3 minutes immersion in methanol, air-dried and stained by a 20–30 minutes immersion in commercial Giemsa diluted with PBS pH 6.8 (1∶2). Identification and counting of different types of white blood cells were done with a light microscope oil immersion lens (1000X). Five types of WBC were considered: heterophiles, eosinophiles, basophiles, lymphocytes and monocytes. For every blood smear, we calculated the proportions of every cell type after 250 cells had been counted. However, we also recorded counts after 100 and 150 cells to obtain repeatability estimates for future studies.

The ratio heterophiles/lymphocytes (H/L) is often used as an indication of stress [34]–[36]. It relies in the known fact that glucocorticoids –the hormonal response to stressor- induces a migration of lymphocytes (L) out of the vascular compartment while enhance the entrance of new heterophiles (H) in blood [37]. We calculated the ratio H/L when 250 WBC were counted in blood.

### WBC Isolation

Our isolation procedure was based in procedures already described [11], [38], [39]. Briefly, whole blood was diluted (1∶1, vol:vol) in Roswell Park Memorial Institute 1640 medium with Hepes (RPMI, Sigma, St. Louis, MO) containing 1% bovine serum albumin (BSA, Sigma), and penicillin-streptomycin−neomycin (200U–0,2 mg-0,4 mg/ml, respectively, Sigma), (RPMI+) and mixed gently. It was set above an equal volume of Histopaque 1.119 (Sigma) and centrifuged at 700 g/30 minutes. The layer above the Histopaque containing the WBC was collected and transferred to a clean tube containing 400 µl RPMI+ and centrifuged at 250 g/12 min. The supernatant was aspirated and the washing procedure repeated. The final cell pellet was gently resuspended in 100 µl of RPMI+ (aspirating and discharging cells with an automatic pipette at least 15 times so that cellular aggregates disaggregate). A 15 µl aliquot was mixed with 5 µl tripan blue and the number of live cells was counted in at least 16–32 small squares in a Thoma chamber. After correcting for dilution, the number of live cells per mm^3^ of cell suspension was calculated. An additional amount of 135 µl of RPMI was added. Only when cell concentration in this suspension was 6,500 –8,500cel/µl the next procedure was used as follows (with values lower than 4,000cel/µl, only 10 µl of the bacterial suspension was used in the next procedure; see below).

### Phagocytosis assay

The phagocytosis assay used is based on the ingestion of fluorescent bacteria and their detection by flow cytometry. To measure the phagocytic capacity of monocytes and heterophiles we used a Fagotest ® kit that uses fluorescein labelled-unopsonised *E Coli* bacteria as phagocytic susceptible material. The commercial procedure was followed with minor modifications (longer time of incubation, 30 min instead 10, and 41°C instead 37°C) and using not whole blood (that contains RBC nucleus as contaminant for flow cytometry) but isolated WBC. Flow cytometry analysis was performed at the Cytometry and Fluorescence Mycroscopy Center of the UCM (Universidad Complutense de Madrid) with a FACScan (Becton Dickinson) cytometer (excitation 488 nm) to measure the percentage of cells with ingested bacteria and the number of bacteria ingested. Data were collected from at least 10,000 cells per sample in the gate set for PMNs, and analysis was done using CellQuest software (BD Biosciences). Results are reported as percentage of either monocytes or granulocytes phagocytosis, since the system does not differentiate the different types of granulocytes. Being heterophiles the only granulocyte type with phagocytic capacity, we refereed to heterophiles phagocytosis in the discussion and abstract sections. This method of measurement of phagocytosis affords a satisfactory repeatability in analysis samples [40].

### Swelling assay: Response to the PHA Challenge

Each nestling was injected at a marked site on the wing web with 0.05 ml of a 5 mg/ml solution of PHA (Sigma, L8754) suspended in phosphate buffer solution (PBS). We measured web thickness to the nearest 0.01 mm at the injection site, using a pressure-sensitive dial thickness gauge (Mitutoyo, Japan). Web thickness was measured twice just before injection and 24 h after it. We calculated the response to the PHA challenge as the change in average thickness (in mm) at the injection site after 24 h [15].

### Data analysis

**Figure 1:**
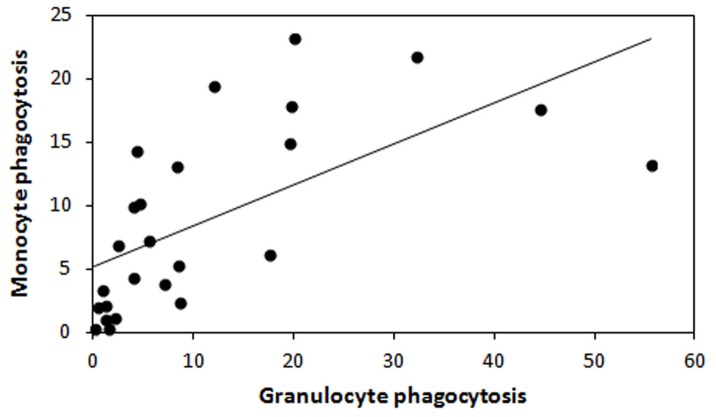
Regression line between monocytes and granulocytes phagocytosis of 14 days-old nestlings spotless starling.

**Figure 2:**
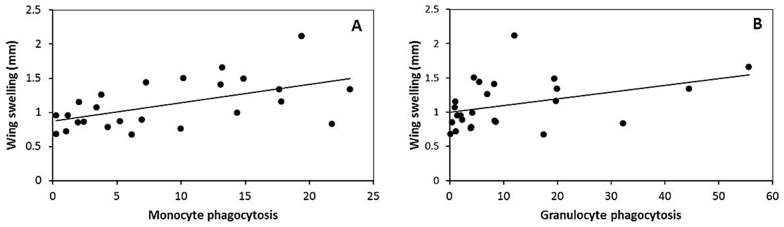
Regression lines between phagocytosis and wing swelling response to PHA injection of 14 days-old nestlings spotless starling. (A) monocyte phagocytosis and (B) granulocyte phagocytosis.

The distributions of variables were tested for normality using one-sample Kolmogrov–Smirnov tests. Whereas hatching date, body weight, RBC numbers, monocyte phagocytosis, and PHA swelling response showed normal distribution (K-S_31_>0.094, *P*>0.200) WBC number, differential counts, H/R ratio, and granulocytes phagocytosis showed highly skewed distributions (K-S_31_<0.094; *P*<0.023) and were logarithmically transformed before analysis. To facilitate interpretation of the results, the plots of granulocyte phagocytosis (Fig. 1 and Fig. 2B) were made with raw data.

We performed linear regressions to evaluate the correlations when counting 250, 150 and 100 cells in blood smears. A paired t-test was performed for testing whether or not PHA injection increased significantly wing thickness. A one-way ANOVA with sex as factor was used to analyse possible sexual differences in both types of phagocytosis and the PHA swelling response. To evaluate the relationship between monocytes and granulocytes phagocytosis, we performed a linear regression with both variables. We built General Linear Models to analyse whether hatching date, body weight, PHA swelling response and nestlings sex may influence both monocytes and granulocytes phagocytosis. Hatching date, body weight and the PHA swelling response were included in initial maximal models, as well as their interactions with nestlings sex. We also built General Linear Models with cell type composition and the H/L ratio as predictors of monocytes phagocytosis, granulocytes phagocytosis and wing swelling. Models were simplified by removing non-significant terms only if AIC values were found to decrease. Statistical analyses were performed in IBM SPSS 20.0.

## Results

Hematological values are depicted in Table 1. Data showed very high correlations for all cell types, regardless of whether 100, 200 or 250 cells were used for the estimation (Table 2). Regressions between data obtained using the smallest and the largest sample (100 vs. 250 cells respectively) were very high for all cell types, with the exception of basophiles which, being the rarest of all cell types, showed a lower, albeit highly significant relationship (Table 2).

**Table 1:** Numbers of RBC, WBC, differential counts and H/L ratio in blood of 14 days old nestlings of spotless starling.

	(cells/mm^3^)		%	
	Mean	SE		SE
RBC	3,801,290	172,206		
WBC	104,983	20,860		
Heterophiles	18,048	3,324	19.60	1.43
Eosinophiles	14,333	2,903	14.74	1.54
Basophiles	143	89	0.28	0.08
Lymphocytes	68,135	14,243	61.45	1.99
Monocytes	4,282	1,074	3.84	0.49
H/L ratio	0.39	0.05		

**Table 2:** Correlations when counting 250, 150 and 100 cells in blood smears for defining the differential counts in blood of 14 days-old nestlings spotless starling.

Cell types	Intraclass correlation	Regression statistics				Regression equation	
		r^2^	*F*	*d. f.*	*P*	slope (SE)	Constant (SE)
Heterophiles	0.919	0.85	156.4	1, 26	0.0001	0.82 (0.07)	3.14 (1.45)
Eosinophiles	0.937	0.85	162.9	1, 26	0.0001	0.85 (0.07)	2.89 (1.15)
Basophiles	0.929	0.52	30.7	1, 26	0.0001	0.51 (0.09)	0.16 (0.04)
Lymphocytes	0.929	0.82	125.59	1, 26	0.0001	0.78 (0.07)	13.1 (4.4)
Monocytes	0.926	0.77	87.83	1, 26	0.0001	0.86 (0.09)	0.43 (0.04)

The number of cells that contained phagocytic material range from 0 to 25% in monocytes while for granulocytes the range was 0–60%. There was a 4 fold increase in wing thickness before (mean ± SE = 0.37 ± 0.45) and after (mean ± SE = 1.49 ± 0.34) of PHA administration statistically significant (paired t-test: t_1, 30_ = −18.537, *P*<0.001).

No sex differences were found in both phagocytosis assays (ANOVAs, monocytes: *F*_1,21_ = 0.001, *P* = 0.973; granulocytes: *F*_1,21_ = 0.019, *P* = 0.890)and in wing swelling (*F*_1,26_ = 0.063 *P* = 0.804). Monocytes phagocytosis was significantly and positively correlated with granulocyte phagocytosis (linear regression, *F*_1,22_ = 28.49, *P*<0.001, corrected *r* ^2^ = 0.56; Fig. 1). Only the PHA swelling response was related with both types of phagocytosis (monocytes phagocytosis: *F*_1,23_ = 9.46, *P* = 0.005, corrected *r* ^2^ = 0.29; Fig. 2A; granulocytes phagocytosis: *F*_1,22_ = 4.41, *P* = 0.047, corrected *r* ^2^ = 0.13; Fig. 2B), but neither hatching date nor body weight were related with phagocytosis (Table 3). Sex was introduced as a factor in the models, but its effects or its interactions were non-significant and thus they were removed from the models. Backward stepwise deletion of terms showed that no measure of phagocytosis or wing swelling response was related to the proportion of any WBC type (all final regressions: *F*_1,22_<1.30, *P* = 0.267).

**Table 3:** General Linear Models relating hatching date, body weight and PHA swelling response to monocytes and granulocytes phagocytosis.

	Standard coefficients		t	P
	B	S.E.		
Monocytes phagocytosis				
Hatching date	−0.31	0.83	−0.38	0.709
Body weight	−0.05	0.42	−0.11	0.911
PHA swelling response	10.86	3.53	3.08	0.005
Granulocytes phagocytosis				
Hatching date	0.11	0.07	1.66	0.112
Body weight	0.01	0.03	0.40	0.701
PHA swelling response	0.64	0.31	2.10	0.047

## Discussion

This study is, to our knowledge, the first one showing a positive relationship between the PHA swelling response and the phagocytic activity in birds. We have found a direct relationship between phagocytosis of both monocytes and heterophiles and the skin swelling response to PHA, i.e., the greater the phagocytosis, the greater the swelling response. As the innate immune response has been assessed by means of the phagocytic activity and also probably by the PHA test and the acquired immune response by means of PHA test and both responses were positively related, this study shows a direct interconnection between both branches of the immune system. Given the correlational nature of our study we are unable to distinguish whether such association is due the PHA response involving both innate and acquired components of the immune system or because individuals with higher innate immunocompetence (before PHA injection) are also more prepared to mount an acquired T-cell response.

It has been previously shown that the molecular form of PHA that produces more intense swelling (PHA-E) is the one that produces the greater tissue damage and the greater number of macrophages and heterophiles in the swelling point [29]. Our results show that when monocytes (the blood cells precursors of tissue macrophages) and heterophiles are confronted with foreign material *in vitro* (*E. coli* bacteria in phagocytic analysis), their phagocytic activity (assessed by the number of cell phagocytizing foreign material) mirrors the swelling response driven at the PHA injection point. Previous studies have shown that the WBC types that increase in number in the first 24 hours after the injection are macrophages and heterophiles (and basophiles, but they are not phagocytic) [20], [29]. Therefore, present results relate the PHA swelling response with the activity of both monocytes and heterophiles. Since these WBC types are principal components of the innate immunological system, this could mean that the PHA injection may also recruit cells of the innate immunological system to the injection point where they fight the foreign or damaged material by phagocytising it. Previous studies claim that the 24 hours swelling response to PHA is representative of the cell-mediated acquired immunity system [8], [22]–[24]. Whether or not the proliferation of T lymphocytes had begun at the time of measuring swelling cannot be evaluated with present results. However, they aim that wing swelling response may not be attributed exclusively to T lymphocytes proliferation. It should be noted that time scale is important when interpreting data from this kind of studies. T-lymphocytes proliferation is measured *in vitro* [4] and is only detected after 48–72 hours of incubation [41]. On the contrary, swelling response is measured *in vivo* 24 hours after injection, when no detectable proliferation takes place *in vitro* [41] (Puerta et al., unpublished data) and the one *in vivo* remains undetermined. Data presented here demonstrates that the PHA test is a valuable tool as long as it is used as a surrogate to evaluate immunocompetence in field studies. Traditionally, the PHA test has been considered as representing the proliferative capacity of T lymphocytes, an event that is ascribed to the acquired or adaptive immunological system [15]. This study shows the involvement of heterophiles and monocytes phagocytosis, events that are ascribed to the innate immunological system. Using the defence component model proposed by Schmid-Hempel and Ebert [10], the acquired component, in immunological terminology, means an induced and specific response in ecological terms whereas the innate component means a constitutive and nonspecific response. Therefore, the involvement of the innate component in the PHA swelling response widens the immunological scope of the PHA test thus reinforcing its use as a tool for immunocompetence studies.

Other possible interpretations may be given from the positive correlation between phagocytic activity and swelling response to PHA. It is known that the magnitude and quality of the acquired immune response is dependent on signals derived from the innate response [42]. There are overlapping and connecting molecules (e.g., cytokines) and cells (e.g., macrophages) that integrate the two branches of the immune system [43]. A primary acquired immune response generally depends on previous activation and participation of the innate immune system, for this reason it is expected that individuals with a greatest development of innate immune system may have a greatest acquired response.

Since no age effects were recorded in the amount of monocytes and heterophiles at the PHA injection point [20] and their phagocytic capacity do not decrease in adults of several species [44], [45], the results we have obtained may be expected in other species and different aged groups.It is important to point out that the phagocytic activity in our study was lower (no more than a 25% for monocytes and a 60% for granulocytes; see Fig. 2A and Fig. 2B) than the one obtained for mammals with the same procedure (commercial protocol; [40], [46]). Phagocytic activity depends upon previous opsonisation of engulfed particles what enhances phagocytosis [31], [44], [45]. The Fagotest® kit uses unopsonised bacteria and it was developed for human use. This means that not only, i. e. RBC are lysed along the procedure not leaving nucleuses that otherwise would interfere with WBC detection in flow cytometry but also that WBC remain in plasma throughout the whole procedure so that *E coli* particles can be oponised. A similar approach cannot be done with bird blood since avian RBC are nucleated. Thus, we isolated WBC to eliminate RBC thus removing plasma this probably leading to the reduction in phagocytic capacity that we have found as alerted by the manufactured and previous studies [31], [44], [45].

We did not find any relationship between hatching date or body weight with any kind of phagocytosis. It seems to disagree with previous studies showing a negative relationship between hatching date and immunocompetence, the latter being judged by the PHA test response and by plasma immunoglobulins Y concentration. Such a negative relationship was related to lower food availability as season proceeds [47], [48]. In fact, such studies found that nestlings hatched early in the season had a higher PHA swelling response than those hatched later. However, in both studies nestlings hatched during a large period, more than one month, time enough to observe food scarcity with the progress of the season. Our study considered chicks that hatched in no more than 9 days, a too short period of time to appreciate a food availability reduction. Accordingly, the disagreement among studies is only apparent. Our nestlings blood contained 61% lymphocytes and 20% heterophiles giving an H/L ration of 0.39 (Table 1) which seems to follow the described pattern of development without the presence of bacterial infections [41], [47]. The nestlings of our study showed a very high proportion of eosinophiles (15%) in agreement with the 22% reported for free-living tree swallow nestlings (*Tachycineta bicolor*; [41]). In the past we also found high percentage of eosinophiles in nestlings of white stork (*Ciconia ciconia*; 22%; [49]) and black stork (*Ciconia nigra*; [50]), in fact, they were much higher than in adults of the same species [49], [51], [52]. High eosinophiles values suggest the presence of parasites in the nesting materials. In fact, an ectoparasite attack recruits both basophiles and eosinophiles to the ectoparasite-feeding point, thus conferring resistance to the ectoparasite so that in a second attack, lower numbers of ectoparasites are successful in proper feeding and therefore have a poorer reproductive success [53], [54]. The nestlings of our study and those in the other cited studies [41], [49] are altricial raising the probability of ectoparasites in the nestling material, as shown in the nests of other species with similar kind of growth [55]–[57]. This could be the reason of the high proportion of eosinophiles in our nestlings. Mean total WBC were about 105×10^3^/mm^3^ in nestlings in our study. This number is larger than in nestlings of other altricial avian species similar in size, like tree swallows (10×10^3^ WBC/mm^3^; [41]) and welcome swallows (*Hirundo neoxena*; 8×10^3^ WBC/mm^3^; [58]) and of those much bigger in size, like storks (60×10^3^ WBC/mm^3^; [49]), common cranes (*Grus grus*; 11×10^3^ WBC/mm^3^; [50]) or flamingos (*Phoenicopterus ruber*; 10–15×10^3^ WBC/mm^3^; [59]). Again, the reason remains uncertain but body size does not appear as a determinant of WBC number. Although WBC number is the result of the state of development and of activation of the immunological system, it does not provide an insight of the effectiveness of every component of the immunological system. Certainly, we did not find any relationship between the number of the different types of WBC and the functionality of the phagocytic cells, either monocytes and heterophils, assessed by a phagocytosis assay.

An additional question posed in this study was to check whether WBC differential counts obtained by counting 250 WBC blood smears were similar to those obtained by counting 100 or 150. This is important since manual counting of blood smears, as needed in species not routinely devoted of veterinarian care, can be tedious and time consuming. Pioneer works recommended a minimum of 400 white blood cells to reach reliable results to use for diagnostic analyses [60]. At present, the number of white blood cell counted is often no more than 100 [35], [36]. To clarify these discrepant criteria we measured 250, 150 or 100 cells per blood smear. Given the correlation between data obtained with 100, 150 and 250 we are confident that counting 100 WBC in blood smears in enough to obtain repeatable differential counts (Table 2).

Finally, the number of RBC were close to 3.8 million for mm^3^ (Table 1) which is higher than those recorded for nestlings of other passerines, namely, welcome swallow (*Hirundo neoxena*) and fairy martin (*Petrochelidon ariel*), that have about 2×10^6^ RBC/mm^3^ [61]. No obvious relationship with body size and its specific metabolic rate seems to be the reason of the difference, taking in consideration that chicks with much bigger body mass, white and black storks [49], flamingos [59] and bustards (*Chlamydotis undulate*; *Eupodotis senegalensis*; *Eupodotis ruficrista*; [62]) have about 2×10^6^ RBC/mm^3^). Factors that have not been evaluated here, as cell volume, haemoglobin content or haemoglobin oxygen affinity could affect those differences in RBC numbers.

In conclusion, we have found for the first time a direct relationship between phagocytic activity and PHA swelling response in birds, suggesting an interconnection between different branches of the immune system. PHA skin swelling response has typically been related to the T lymphocyte proliferation, an acquired immunological process [63], [64]. However, present evidence suggests that it is also related to phagocytosis -an innate immunological process. Whatever the branches of immunological system are being assessed with the PHA skin swelling test, our study highlights that the PHA test is a reliable indicator of immunocompetence.
